# Propylene glycol and Kolliphor as solvents for systemic delivery of cannabinoids via intraperitoneal and subcutaneous routes in preclinical studies: a comparative technical note

**DOI:** 10.1186/s42238-023-00194-9

**Published:** 2023-06-20

**Authors:** Kaveh Momenzadeh, Diana Yeritsyan, Nadim Kheir, Rosalyn M. Nazarian, Ara Nazarian

**Affiliations:** 1 Musculoskeletal Translational Innovation Initiative, Beth Israel Deaconess Medical Center, Harvard Medical School, Boston, MA 02115 USA; 2 Pathology Service, Dermatopathology Unit, Massachusetts General Hospital, Harvard Medical School, Boston, MA USA; 3 Department of Orthopaedic Surgery, Yerevan State Medical University, Yerevan, Armenia

**Keywords:** Cannabinoids, Route of administration, Drug delivery, Animal models, Skin histopathology

## Abstract

**Background:**

Substance administration to laboratory animals necessitates careful consideration and planning in order to enhance agent distribution while reducing any harmful effects from the technique. There are numerous methods for administering cannabinoids; however, several parameters must be considered, including delivery frequency, volume of administration, vehicle, and the level of competence required for staff to use these routes properly. There is a scarcity of information about the appropriate delivery method for cannabinoids in animal research, particularly those that need the least amount of animal manipulation during the course of the investigation. This study aims to assess the feasibility and potential side effects of intraperitoneal and subcutaneous injection of CBD and THC using propylene glycol or Kolliphor in animal models. By evaluating the ease of use and histopathological side effects of these solvents, this study intends to help researchers better understand an accessible long-term delivery route of administration in animal experiments while minimizing the potential confounding effects of the delivery method on the animal.

**Methods:**

Intraperitoneal and subcutaneous methods of systemic cannabis administration were tested in rat models. Subcutaneous delivery via needle injection and continuous osmotic pump release were evaluated using propylene glycol or Kolliphor solvents. In addition, the use of a needle injection and a propylene glycol solvent for intraperitoneal (IP) administration was investigated. Skin histopathological changes were evaluated following a trial of subcutaneous injections of cannabinoids utilizing propylene glycol solvent.

**Discussion:**

Although IP delivery of cannabinoids with propylene glycol as solvent is a viable method and is preferable to oral treatment in order to reduce gastrointestinal tract degradation, it has substantial feasibility limitations. We conclude that subcutaneous delivery utilizing osmotic pumps with Kolliphor as a solvent provides viable and consistent route of administration for long-term systemic cannabinoid delivery in the preclinical context.

## Introduction

The effects of cannabis vary depending on the formulation, dose, concentration, and route of administration (Schwotzer et al. 2022; Hlozek et al. 2017; Grotenhermen 2003; Huestis 2007). Inhalation, oral, aerosol, transdermal, subcutaneous, rectal, sublingual, intravenous, intraperitoneal, and ocular are all available routes of administration for cannabis products. In clinical and recreational settings, the most common routes of administration for cannabis vary depending on the purpose and context. Inhalation (smoking or vaporizing) and oral ingestion are often preferred methods due to their rapid onset of effects and ease of use (Grotenhermen 2003; Mechoulam et al. 2007; MacCallum and Russo 2018). These routes allow for efficient delivery of cannabinoids into the bloodstream, leading to psychoactive and/or therapeutic effects. In preclinical studies, there is a wider range of routes of administration used to investigate the effects of cannabinoids. While inhalation and oral routes are still commonly employed, other routes such as intravenous (IV), intraperitoneal (IP), subcutaneous (SC), and transdermal are also utilized depending on the research objectives and animal models (Hlozek et al. 2017; Valvassori et al. 2013; Nelson et al. 2019; Fried 1976; Rock et al. 2016; Klein and de Quadros De Bortolli J, Guimaraes FS, Salum FG, Cherubini K, de Figueiredo MAZ. 2018; Madularu et al. 2017). These routes offer different advantages in terms of pharmacokinetics, bioavailability, and target organ/system distribution, and it is crucial in optimizing cannabis’ therapeutic potential while mitigating potential risks and adverse effects.

With the recent wave of legalizations, permitting medical use in 37 states, and recreational use in 21 states and Washington, D.C., cannabis consumption is expected to grow, where its use is particularly prevalent among adolescents and young adults (Pacula and Smart 2017). With this in mind, research interests are also increasing to further elucidate the biological, physiological, and behavioral effects of cannabinoids on various organ systems and distinguish factors contributing to individual user-based variations. In the research setting, innovation relies on animal models, where mice and rats are commonly used in studies involving cannabinoids.

The bioavailability of cannabinoids varies significantly depending on the route of administration. Inhalation and intravenous (IV) injection have the highest bioavailability, with approximately 10–56% and 100%, respectively (Grotenhermen 2003; Huestis 2007). This is due to the direct absorption of cannabinoids into the bloodstream, leading to a rapid onset of action. Oral administration has lower bioavailability, ranging from 6 to 20% (Grotenhermen 2003; Huestis 2007), due to first-pass metabolism in the liver and gastric acid degradation. This results in a delayed onset of action and decreased efficacy compared to inhalation or IV injection. Transdermal and rectal cannabinoids routes of administration may also have lower bioavailability, with rectal reported ranging 13.5% and up to twice the oral delivery, although the exact bioavailability for these routes can vary depending on the specific formulation and permeation enhancer used (Grotenhermen 2003; Huestis 2007; Mahmoudinoodezh et al. 2022; Paudel et al. 2010; Stinchcomb et al. 2004). In terms of animal studies, intraperitoneal (IP) injection and subcutaneous (SC) injection are also commonly used routes of administration for cannabinoids. The bioavailability of IP injection has been reported to be 60–90%, while subcutaneous injection is considered to range from 65% up to equivalent to IV injection in terms of bioavailability (Grotenhermen 2003; Al Shoyaib et al. 2019; Nakano et al. 2019). Several studies on the subcutaneous delivery of cannabinoids have reported adverse effects on the subcutaneous tissue, including inflammation, degeneration, and necrosis (Banerjee et al. 1976; Kamali-Sarvestani et al. 2020; Sofia et al. 1979; Thompson et al. 1975).

This study assesses the feasibility and potential side effects of IP and SC injection of CBD and THC using propylene glycol or Kolliphor in animal models. Specifically, the study will evaluate the ease of use and histopathological side effects of the selected solvents for the systemic delivery of cannabinoids in animal models. By providing an evaluation of these alternative approaches, this study intend to help researchers better understand an accessible long-term delivery route of cannabinoids in animal experiments while minimizing the potential confounding effects of the delivery method on the animal.

## Methods

Animal experiments were approved by the local Institutional Animal Care and Use Committee (IACUC) at Beth Israel Deaconess Medical Center. The National Institute on Drug Abuse (NIDA) supplied tetrahydrocannabinol (THC) dissolved in ethanol and cannabidiol (CBD) in powder form. Prior to resuspension, THC was reconstituted in a vacuum dryer to remove the ethanol content. Cannabinoids were delivered systemically through two subcutaneous (SC) routes — injection and osmotic pump (Model 2ML4, ALZET, USA) — and one intraperitoneal (IP) route, using two solvents (propylene glycol and Kolliphor). The studies performed are outlined in Table 1.

**Table 1:** Studies performed

Study	Route	Mechanism of delivery	Solvent/vehicle	Groups (dosage 5 mg/kg)	Frequency of delivery	Euthanasia endpoint	Sample size (per group)
**Study 1**: ALZET osmotic pump subcutaneous delivery (original study identification of issue)	SC	Osmotic pump	Propylene glycol	THC, CBD, CBD + THC, vehicle	Continuous/daily	Early euthanasia	*N* = 4^a^
**Study 2**: ALZET osmotic pump subcutaneous delivery with provisions to reduce risk of contamination	SC	Osmotic pump	Propylene glycol	THC, CBD, CBD + THC, vehicle	Continuous/daily	Early euthanasia	*N* = 2^a^
**Study 3**: Isolated THC, CBD direct subcutaneous injection	SC	25-GA needle, 0.1 ml	THC dissolved in ethanol CBD dissolved in saline	THC (*20 mg/day*) CBD (*20 mg/day*)	Daily	7 days	*N* = 2^a^
**Study 4**: Direct injection subcutaneous delivery, histopathology	SC	25-GA needle, 0.1 ml	Propylene glycol	THC, CBD, CBD + THC, vehicle, saline	Daily	10 days	*N* = 2^a^
**Study 5**: Intraperitoneal delivery	IP	22-GA needle, 0.1 ml	Propylene glycol	THC, CBD, CBD + THC, vehicle	Weekly	8 weeks	*N* = 12
**Study 6**: ALZET osmotic pump subcutaneous delivery with change in solvent	SC	Osmotic pump	1:1:18 parts ethanol:Kolliphor:saline	THC, CBD, CBD + THC, vehicle	Continuous/daily	8 weeks	*N* = 4

^a^Side effects and observations made were seen in all rats ubiquitously

All studies were conducted on 13-week male Sprague Dawley rats (approximately 330 g, Charles River Laboratories, MA, USA. Divided equally within each study arm) with the aim of identifying the optimal route of administration with the least delivery-associated side effects. All surgical procedures were performed under sterile conditions: rats were shaved, and disinfected with a betadine and alcohol mixture at the surgical site, and surgical tools were sterilized. Osmotic pumps were handled under sterile conditions and implanted under general anesthesia using 5% isoflurane for induction and 2.5% for maintenance. The animals were carefully monitored during and after the procedure for any signs of distress or discomfort. All other injections were administered on restrained animals without the use of anesthesia. For all THC and CBD, treatment groups’ dosage was at 5 mg/kg, while the combination CBD + THC group received 5 + 5 mg/kg, respectively, except for study 3, where a higher dosage of 20 mg/kg was used. Issues described in this technical note came about through investigation of the effects of cannabinoids in orthopedic musculoskeletal preclinical animal models of injury such as posterolateral lumbar spinal fusion, intramedullary femoral shaft pinning, or femoral shaft critical size bone defect, where repetitive animal handling for agent delivery could potentially endanger fracture healing progression.

## Results

**Fig. 1:**
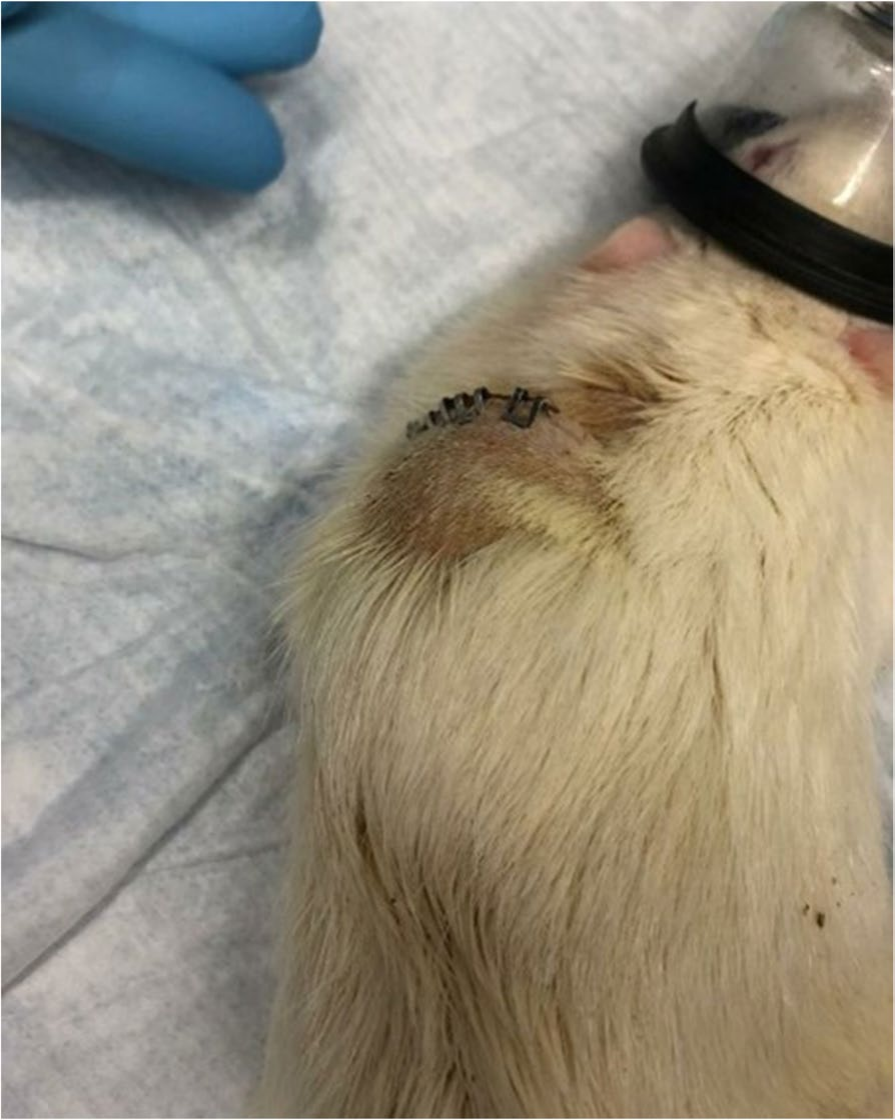
Erythema, inflammation, and fluid accumulation around the pump

Initially, we observed no immediate postoperative swelling or erythema at the pump insertion site in *Study 1*. Our first observations occurred on postoperative day 4, where the skin over the pump and the pump insertion site became erythematic and swollen. Pus was found near the pump (Fig. 1). There were signs of back abrasions thought to be self-inflicted, due to pruritus or an allergic reaction. Intramuscular enrofloxacin was given to treat the swelling and reduce the inflammation. Once the antibiotic was stopped, the swelling returned, and an abscess formed again around the pump. To prevent any further pain and morbidity, the animals were euthanized early.

Bacterial contamination was thought to be the cause based on the results from Study 1. Therefore, *Study 2* was a repeat of Study 1 with four key revisions to reduce the risk of contamination. The vacuum dryer used to resuspend the THC was cleaned with 70% isopropyl alcohol. Pumps were cleaned with 70% isopropyl alcohol before insertion. A new stock solution of propylene glycol was ordered and used for reagent preparation. Finally, 0.22-μm pore size microfilters (MilliporeSigma™ Millex™ Sterile Syringe Filters, USA) were used to filter the prepared reagents of microorganisms before filling the sterile pumps. Despite these steps, we observed the same adverse reaction to pump placement, confirming causes other than contamination as the source. Similarly, the animals were euthanized early to prevent suffering.

After ruling out microbial contamination as the source of the adverse effects, in Study 3, we sought to identify if the NIH-provided cannabinoid agents were the cause of irritation. This was achieved through direct subcutaneous injection of the agents with minimal processing and elimination of additional confounders. THC dissolved in ethanol (as supplied by the NIH) and CBD dissolved in saline (NIH-provided powder form) were used. Both THC and CBD injections resulted in cutaneous ulcers at the injection site.

**Fig. 2:**
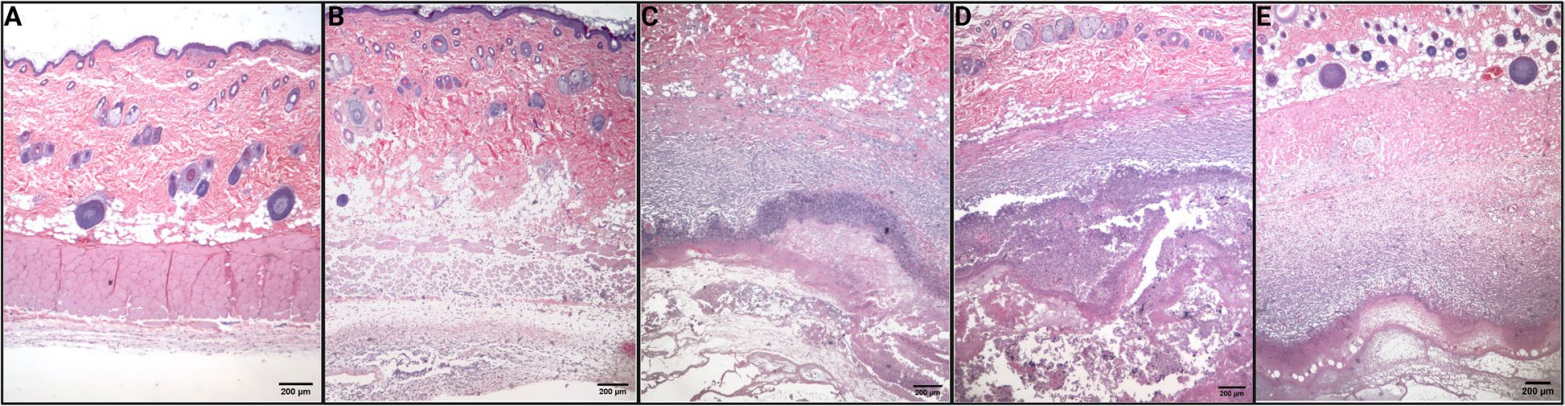
H&E staining of skin samples from treated rats. **A** Saline. **B** Propylene glycol. **C** CBD only. **D** THC only. **E** CBD + THC. **A** Saline: normal skin, no significant inflammation. **B** Propylene glycol: extensive superficial and deep neutrophilic inflammation with associated granulation tissue. **C** CBD only: ranging from granulation tissue and palisading neutrophilic abscess with necrobiosis, debris, and surface bacterial infection to ulcer and full-thickness cutaneous necrosis. **D** THC only: granulation tissue and palisading neutrophilic abscess with necrobiosis and debris. **E** CBD + THC: granulation tissue and palisading neutrophilic abscess with necrobiosis and debris

Having only observational data thus far, we conducted *Study 4* by direct injection of THC, CBD, THC + CBD, vehicle (propylene glycol), and saline subcutaneously to assess the skin histopathology of cannabinoids injections, delivered via the conventional solvent. In rats receiving THC-only injections, erythema was observed at the injection site immediately after injection, and a liquefaction abscess formed 3 days post-injection. Ten-day skin H&E histological stains (Fig. 2) showed granulation tissue and palisading neutrophilic abscess with necrobiosis and debris in all three cannabinoid groups. CBD-exposed skin also had a surface bacterial infection along with an ulcer and full-thickness cutaneous necrosis in one of the samples. Propylene glycol-only-treated samples showed either an ulcer with superficial and deep neutrophilic inflammation or extensive chronic inflammation of the muscle layer with granulation tissue. Normal skin was observed after saline exposure.

Following these studies, specifically considering observations from Study 3, where direct isolate THC and CBD injections caused a reaction, and Study 4, where we observed histological evidence of skin changes, in *Study 5*, we shifted to intraperitoneal (IP) delivery with the same groups and solvent as those used in Study 1. We observed no immediate erythema, induration, pruritus, pain, warmth, or any delayed effects of rash, formation of subcutaneous nodule or granuloma, or ulceration in the animals in this group.

Given the known difficulties with IP injections in rodents, the additional distress on the animals, and reduced bioavailability, we returned to osmotic pump-based subcutaneous delivery, this time with a different solvent. In *Study 6*, we adopted a new solvent, Kolliphor, predominantly dissolved in saline, as a solubilizer and emulsifier, thus shifting from a hydrophobic composition to a hydrophilic composition. We observed no irritation or dermatological reactions in this group throughout the study. The animals were monitored for 8 weeks, with a pump replacement at 4 weeks, and no erythema or skin abnormalities were noted during the experiment. The pumps and subcutaneous area were examined for signs of infection or abscess formation during the pump replacement at 4 weeks and found to be clear.

## Discussion

The route of administration of drugs in a research setting is determined by the study needs and experimental conditions, ease of administration, the drug’s characteristics, and the adverse effects related with the delivery route/technique, all informed by the desired effects, efficacy, therapeutic window, and drug effect onset and offset, among other considerations. Cannabinoids are being investigated extensively in preclinical investigations as their receptors are widely distributed throughout the body. A route of administration with the least amount of animal manipulation is advised, especially for studies where multiple animal handling may influence the outcome of the investigation, such as in musculoskeletal research.

Our studies were performed in the musculoskeletal research setting. According to our findings, propylene glycol, a conventionally excellent hydrophobic/lipophilic solvent, may potentially cause synergistic or at least additive adverse effects when utilized as a cannabinoid vehicle for subcutaneous injection. Our findings were consistent with those reported by Banerjee et al. and Thompson et al., who observed comparable skin reactions in rabbits (Banerjee et al. 1976; Thompson et al. 1975).

IP injections have previously been used in several studies, including those by Harte et al., Moreira et al., and Craft et al. (Altinok et al. 2015; Beydogan et al. 2019; Craft et al. 2012; Harte and Dow-Edwards 2010; Moreira et al. 2006). Various solvents such as DMSO (Ahmadi et al. 2021; Calik and Carley 2017; Dajani et al. 1999; Sheerin et al. 2004), Emulphor (Welch et al. 1998; Wiley and Burston 2014), polysorbate 80 with saline (Borgen and Davis 1973; Marusich et al. 2014), and Cremophor also known as Kolliphor (Moore et al. 2021; Ozaita et al. 2007) have been used to dissolve THC and other cannabinoids receptor ligands, though do not mention using these formulations with CBD. The efficacy of long-term continuous cannabinoid administration with these solvents is also unclear, as most studies look at short-term single injections. Still, there is potential for them to be effective in long-term studies, as seen in Ahmadi et al. 2021 (DMSO) and Moore et al. (2021) (Cremophor) where they used THC for a duration of 21 days and 14 weeks, respectively (Ahmadi et al. 2021; Moore et al. 2021). However, there are drawbacks associated with IP therapeutic administration, such as first-pass hepatic metabolism, which reduces the bioavailability of the treatment agents (Al Shoyaib et al. 2019). Moreover, as IP injections are typically performed in conscious animals using firm manual restraint, they are associated with stress for both the staff and the animal, and even competent staff 20% of the time deliver IP injections in areas other than the peritoneum such as the gastrointestinal tract, subcutaneously, retroperitoneally, or into the urinary bladder (Morton et al. 2001; Lewis et al. 1966; Turner et al. 2011; Zatroch et al. 2017). On the other hand, SC injections are generally considered a more feasible and less stressful route of administration. SC injections are typically performed in the loose skin between the animal’s shoulder blades or along the side of the animal and do not require extensive restraint or manipulation of the animal (Turner et al. 2011). With these limitations in IP delivery and our observations of subcutaneous propylene glycol delivery, we investigated Kolliphor as a new solvent for THC and CBD delivery (1:1:18 parts ethanol:Kolliphor:saline). These have been used successfully with ALZET pumps to deliver cannabinoids in previous studies by Zimmer et al., who suggested massaging the pump for the first three postoperative days (Nidadavolu et al. 2021).

## Conclusion

Our studies with subcutaneous and pump delivery of cannabinoids, suspended in Kolliphor, demonstrated that subcutaneous delivery without any delivery site dermatologic reactions is possible, one that will bypass hepatic metabolism as well. Subcutaneous delivery with Kolliphor also obviates the need for multiple injections. Although IP administration is favored over oral administration for biological agents to avoid gastrointestinal tract degradation, it has considerable feasibility drawbacks. We conclude that among the routes and solvents tested in the current study, osmotic pumps with Kolliphor as a solvent present the best route of administration for sustained systemic delivery of cannabinoids in the preclinical setting.

## Data Availability

Data sharing is not applicable to this article as no datasets were generated or analyzed during the current study.

## Abbreviations

IACUC: Institutional Animal Care and Use Committee
NIDA: National Institute on Drug Abuse
THC: Tetrahydrocannabinol
CBD: Cannabidiol
SC: Subcutaneous
IP: Intraperitoneal
